# IMPROVING DETECTION OF UNTREATED HEARING LOSS IN A COMPREHENSIVE CARE SETTING USING A SINGLE-QUESTION SCREENER

**DOI:** 10.1093/geroni/igae098.2532

**Published:** 2024-12-31

**Authors:** Sara Mamo, Alyssa Krysko, Katherine Garrity, Margaret Wallhagen

**Affiliations:** University of Massachusetts Amherst, Amherst, Massachusetts, United States; University of Massachusetts Amherst, Amherst, Massachusetts, United States; University of Massachusetts Amherst, Amherst, Massachusetts, United States; University of California San Francisco, San Francisco, California, United States

## Abstract

A majority of older adults have untreated hearing loss, which poses care and communication challenges for healthcare providers. Routine identification of hearing loss would help providers and individuals recognize hearing as a possible contributor to communication difficulties. The objective of the study was to implement a single-question hearing screening administered by medical assistants (MAs) in a health clinic. The study took place in a Day Health Clinic at a Program for All-Inclusive Care for the Elderly (PACE). Participants included anyone seen in the clinic during the study. Participants were screened by the MA, and the researchers collected hearing thresholds after their appointment. We performed behavioral hearing threshold testing and used a 4-frequency pure-tone average to estimate the individual’s hearing status and measure the sensitivity/specificity of the screening method. With a sample of 49 participants (age range = 56-90 years), we calculated a sensitivity of 71.4% and a specificity of 42.9%. Categorically, our behavioral hearing tests resulted in 14 individuals with clinically normal hearing, 24 with mild hearing loss, and 11 with moderate or worse hearing thresholds. There were no current hearing aid users in the sample, and three who reported having used hearing aids previously. The findings suggest that a single-question screener can be used as a simple method of identifying individuals who may benefit from a hearing evaluation and/or hearing loss treatment. Future studies should integrate ‘hearing status’ prominently in the individual’s medical record and establish protocols for using personal amplifiers to support adults with untreated hearing loss.

